# Frogs’ Legs Versus Roast Beef: How Culture Can Influence Mind-Wandering Episodes Across the Lifespan

**DOI:** 10.5964/ejop.v15i2.1597

**Published:** 2019-06-07

**Authors:** Léa M. Martinon, Jonathan Smallwood, Colin Hamilton, Leigh M. Riby

**Affiliations:** a Psychology Department, Northumbria University, Newcastle-upon-Tyne, United Kingdom; b Psychology Department, University of York, York, United Kingdom; Webster University Geneva, Geneva, Switzerland; Maria Grzegorzewska University, Warsaw, Poland

**Keywords:** mind-wandering, day-dreaming, cultural differences, self-generated thoughts, aging, rumination, reflection

## Abstract

Numerous studies have captured the nature of mind-wandering and how it changes across the lifespan; however, the influence of culture has been neglected. This study investigated the joint effects of culture and age in a large scale online questionnaire-based survey of 308 adults over 18 years of age, both in France and the United Kingdom. To capture a profile of thinking style, self-report measures of mind-wandering frequency, mindfulness, mood, rumination, self-reflection, future thinking, depressive symptoms, and cognitive failures were gathered. Findings revealed an earlier decrease in mind-wandering frequency for French speaking participants. Cultural effects were demonstrated on rumination and reflection rates across the life span, with in general more rumination and less reflection for English speakers. Overall, negatively toned thoughts were dominant for English compared to more expressive thoughts in general for French speakers. Confirmatory factor analyses featured different theoretical models to explain mind-wandering frequency in the French and British populations. This study provides the basis for further investigations of sociocultural influences on the eclectic phenomenon of mind-wandering.

The study of the wandering mind has accelerated in recent years, primarily due to the importance of such thought processes on everyday behaviour and wellbeing. In the literature, mind-wandering has been referred to as task-unrelated thought (Giambra, 1989), daydreaming (Cundiff & Gold, 1979; Poerio, Totterdell, Emerson, & Miles, 2015), and more recently self-generated thought (Smallwood, 2013). The commonality between all is the disengagement from the processing of the external world to internal thoughts and feelings. However, trains of thought of this nature can take many forms (i.e., related to the self or to others, to the past, or to the future; Baird, Smallwood, & Schooler, 2011) and have both positive and negative consequences (e.g., planning and enhanced problem solving, Baird et al., 2012; cognitive failures, Mooneyham & Schooler, 2013; and mood changes, Poerio, Totterdell, & Miles, 2013). Mind-wandering is known to take up anywhere from a third to half of our mental life (Killingsworth & Gilbert, 2010), and can impact on everyday life activities (Cowley, 2013; McVay, Kane, & Kwapil, 2009) yet little is known about how culture impacts on the frequency, content, and consequences of mind-wandering across the lifespan. The phenomenon has been investigated across the globe; namely, China (Deng, Li, & Tang, 2012; Song & Wang, 2012), United States of America (Levinson, Smallwood, & Davidson, 2012), United Kingdom (Smallwood, Nind, & O’Connor, 2009), Belgium (Stawarczyk, Majerus, Maj, Van der Linden, & D’Argembeau, 2011) and Germany (Tusche, Smallwood, Bernhardt, & Singer, 2014). Yet, given how socio-cultural research has dominated investigations into psychological phenomenon, it is surprising that no recent direct comparisons of populations on the nature and frequency of self-generated thoughts have been carried out.

## Cultural Implications

Very little research has focused on the impact of culture on mind-wandering experiences. One large scale questionnaire study explored characteristics of adults’ daydreaming; Singer and McCraven (1961) evidenced cultural differences in daydreaming frequency and content. Afro-American and Jewish participants presented the highest daydreaming frequency compared to their British counterparts. Although the nature of the variability was not specified, the authors argued that variations in family constellations (complex bonds and family relationships) and life experiences in general drive such differences. Indeed, Jewish cultural values, such as the encouragement of self-reflection and open communication between family members, may play an important role in mind-wandering frequency and identity creation. Elsewhere, research has evidenced that mind-wandering scaffolds and maintains a continuous feeling of “self” and identity (Smallwood et al., 2011; Song & Wang, 2012). Rather than explaining the pattern of findings in terms of relatively more positive aspects of daydreaming (self-reflections), for the Afro-Americans, negative life events (e.g., bullying) were suggested to give rise to a greater propensity for unpleasant thoughts and the likelihood of developing psychological problems associated with rumination (e.g., Depression, Salmon, 2000; Erdur-Baker, 2009). More recently, research showed that European-heritage students tend to mind wander more than Asian-heritage students or Japanese exchange students (Sude, 2015). Yet, others failed to demonstrate any cultural differences, between Portuguese and Brazilian participants, in terms of frequency or content of mind-wandering experiences (Gonçalves et al., 2017). Nevertheless, their results suggested that Portuguese students used mind wandering to their advantage more than Brazilian students. Together these studies point to the influence of culture on the occurrence and the characteristics of mind wandering, and provides the foundation for the study presented here.

Although the examination of cultural differences in mind-wandering has been neglected, data from the language domain are informative and may aid in the generation of hypotheses for future experimental work. Our thoughts and feeling during mind-wandering episodes are composed largely of inner speech rather than visual imagery (Bastian et al., 2017; Stawarczyk, Cassol, & D’Argembeau, 2013). Additionally, once internalized, language plays a crucial role in shaping cognitive processes and organizing thoughts (Vygotsky, 1962). According to Whorf (1956), certain properties of a given language affect the way people perceive and remember and so through language, culture may influence people’s self-generated and unconscious thought processes. For example, the different way of talking about time between English and Mandarin corresponds to discrepancies in how English and Mandarin speakers think about time (Boroditsky, 2001). Abstract thoughts involving time tend to be shaped and organised differently with vertical (Mandarin) and horizontal (English) metaphors driving how time is conceptualized. Therefore, considering that two languages may impact on the nature of abstract thoughts differently, it is not unreasonable to suggest language will have a more universal impact on self-generated thoughts studied here.

The vast majority of studies investigating cultural differences on psychological phenomena often compare western and eastern populations where more striking differences are expected. Comparison within European countries is fairly rare but due to geographic proximity, cultural similarities may be predicted. Yet, some observations suggest clear cultural differences existing between French and British citizens. For example, the literature regarding the “Déjà vu” experience suggests that French speakers describe their experience of such thoughts as more disturbing than English speakers (Fortier & Moulin, 2015). Additionally, a crucial element when investigating cultural discrepancies, is the strong influence of the individuals’ educational background on the development of moral value and shaping of thought processes. The education system is considered to be a product of societal norms shared by the majority of the population (Hofstede, 1984), and as such, the notable differences between the French and British educational system suggest divergent cultural values. The French educational system is mostly focused on knowledge and intellect, whereas the British system mainly focused on the development of the individual (Mons, Duru-Bellat, & Savina, 2012). This difference can be illustrated within disparate professions. For example, in the management environment, French managers show a tendency to be more control orientated, in contrast to their British counterparts that are more prone to delegation and trust (Gröschl & Barrows, 2003). Many more examples of divergent social and psychological styles (e.g., writing methods) between French and British individuals could be highlighted here but overall one could expect more fundamental difference in the nature of thinking in the form of self-generated thoughts.

## Mind-Wandering Characteristics

A growing body of research has explored the variety of content that comprise self-generated thoughts. Recent work has focused on the temporal dynamics of mind-wandering, revealing a tendency for thoughts to be directed toward the future (Baird et al., 2011; D’Argembeau, Renaud, & Van der Linden, 2011; Smallwood, Nind, et al., 2009). Future-oriented thoughts seem to be characterised by a verbal form, with a tendency to be realistic, concrete, structured and more personally relevant (Stawarczyk et al., 2013). Future thinking has been related to recovery from negative responses such as stress (Engert, Smallwood, & Singer, 2014) and are commonly associated with positive mood (Ruby, Smallwood, Engen, & Singer, 2013). Self-reflection is a core component of future thinking during mind-wandering, and self-generated thoughts are more often about the self than others (Baird et al., 2011; Smallwood et al., 2011). While the majority of spontaneous cognition is future oriented, a smaller but significant proportion of off-task thoughts are directed to the past (Baird et al., 2011). Negative mood induction procedures have consistently been found to induce greater past-related thoughts (Smallwood & O’Connor, 2011). In the absence of mood induction, it has been evidenced that sadness tends to precede mind-wandering occurrence, and having negatively toned thoughts during mind-wandering predicts subsequent sadness (Poerio et al., 2013). Overall, thoughts directed toward the past tend to induce negative mood while future and self-related thoughts tend to improve mood.

A further key research endeavour is the exploration of mind-wandering outcomes. If different mind-wandering styles occur across cultures, this could have some bearing on the positive and negative consequences of such episodes. For instance, mind-wandering is reported to have deleterious effects on cognitive abilities including working memory, sustained attention and comprehension (for a review see Mooneyham & Schooler, 2013). Similarly, on everyday activities, engaging in mind-wandering while driving increases error rates and impacts on driver safety (Cowley, 2013; Qu et al., 2015). Mirroring work on mind-wandering, data from the mindfulness and mediation literature is informative regarding impacts on task performance. Mindfulness has been shown to reduce mind-wandering frequency with subsequent improvements on everyday activities (Mrazek, Franklin, Phillips, Baird, & Schooler, 2013). Mindfulness practice is often associated to well-being (Brown & Ryan, 2003) and increased cognitive abilities (Chiesa, Calati, & Serretti, 2011), however mind-wandering also seems to benefit individuals. As mind-wandering is in part goal oriented (D’Argembeau et al., 2011), and mostly refers to the self and the future, then it has been argued that one of the primary functions of mind-wandering is to enable the anticipation and planning of personally relevant future goals (Baird et al., 2011). Additional functions highlighting the adaptive value of mind-wandering have been identified including enhanced social problem solving (Ruby et al., 2013), the fostering of both a more patient style of making decisions (Smallwood, Ruby, & Singer, 2013) and more creative processing whilst problem solving (Baird et al., 2012).

Particularly relevant here, the mind-wandering literature has considered how the ageing process modulates the frequency of mind-wandering. Age-related decreases in cognitive ability have largely been report on those function sub-served by the frontal lobes (e.g., executive control and inhibition) and hippocampus (e.g., episodic memory). Given the widely reported executive function deficit in ageing (Riby, Perfect, & Stollery, 2004a, 2004b), intuitively one would expect greater intrusive thoughts for older individuals due to difficulties in effectively inhibit irrelevant thoughts and feelings. Paradoxically, multiple studies have reported a decrease in the frequency of mind-wandering episodes as one grows older (Giambra, 1989; Jackson & Balota, 2012; Jackson, Weinstein, & Balota, 2013). It is worthwhile noting however that the complexity of the thoughts and specific content of mind-wandering episodes has in the main been neglected. Characteristics that have been associated with the ageing process can give clues to the specific components that influence the nature of mind-wandering episodes. For example, older adults are thought to be more mindful and present moment focussed (Splevins, Smith, & Simpson, 2009) and largely have more positive moods (Carstensen, Isaacowitz, & Charles, 1999). Regarding future thinking, Rendell et al. (2012) showed that older adults were worse at imagining the future than a-temporal experiences. Thus, their frequency of future thoughts decreases (Berntsen, Rubin, & Salgado, 2015) but they also seem to experience more a-temporal than future directed mind-wandering (Jackson et al., 2013). Overall, the nature of self-generated thoughts is somewhat complex during mind-wandering episodes as we age.

## The Present Study

The purpose of this study was to identify broad discrepancies in mind-wandering by examining the frequency between French (France) and English (United Kingdom) native speakers, in young, middle-aged and older adults. In order to further characterise the joint effects of age and culture a collection of seven questionnaires were used to capture key elements previously associated with the wandering mind in the literature. Namely, mindfulness abilities, mood, future thinking, self-attentiveness (rumination and reflection), cognitive failure, and depressive symptoms measures were gathered. Unfortunately, the literature regarding the influence of culture on mind-wandering is lacking, impeding the generation of precise hypotheses, particularly when it comes to two European countries. However, variables capturing mood and emotional content (e.g., rumination vs. reflection) will establish whether a previously identified bias for negatively toned thoughts in English speakers and more expressive thoughts for French speakers (e.g., work examining the Déjà vu experience; Fortier & Moulin, 2015) is also observed for self-generated thought. Regarding the overall effects of age, we anticipate a decrease in mind-wandering frequency, future thinking and depressive symptoms (Berntsen et al., 2015; Giambra, 1989; Nolen-Hoeksema & Ahrens, 2002) alongside an increase in cognitive failures and mindfulness abilities (Mecacci & Righi, 2006; Splevins et al., 2009). In addition, structural equation modelling will be used to generate an overview of the relationships linking mind-wandering experiences to the measures collected. Conceptually, we expect that measures of rumination, negative mood and depressive symptoms will compose a negative construct (Nolen-Hoeksema, Wisco, & Lyubomirsky, 2008; Poerio et al., 2013; Smallwood, O’Connor, & Heim, 2005). Additionally, we expect that the frequency of future-self related thoughts and self-reflection will comprise a self-related construct (Stawarczyk et al., 2013; Trapnell & Campbell, 1999), and that measures of mindfulness and cognitive failures will make an attentional construct (Chiesa et al., 2011; Mooneyham & Schooler, 2013). Ultimately, we hypothesis that the theoretical model, where mind-wandering frequency is predicted by age and the previously outlined constructs (self-related, negative and attentional), will fit the present data. However, once again, the lack of research on the impact of culture on mind-wandering experiences prevents us from formulating clear hypothesis about the variations of the model in the two populations, yet differences are suspected (Gonçalves et al., 2017; Singer & McCraven, 1961; Sude, 2015).

## Method

### Participants

A total of 471 volunteers completed an online survey, 283 in English and 188 in French. Fourteen non-English native speakers were removed from the English data set. One non-French native speaker was removed from the French data set. However, significant differences were found between the samples in terms of age, *F*(1, 453) = 7.56, *p* = .006, $\eta_{p}^{2}$ = .02, and years of educations, *F*(1, 451) = 4.56, *p* = .033, $\eta_{p}^{2}$ = .01. Therefore, we choose to match the sample on those two variables. The remaining data consisted of 154 native English speakers (*M* = 31.21, *SD* = 16.15, 132 female) and 154 native French speakers (*M* = 32.32, *SD* = 15.96, 130 female). To be inclusive as possible, the inclusion criteria were to be 18 or over and be native English or French speakers. This study was approved by the Ethics Committee of the Faculty of Health and Life Sciences of Northumbria University. The investigation was conducted according to the principles expressed in the Declaration of Helsinki and for both populations, participants provided informed consent.

### Measures

#### Mind-Wandering

The Daydreaming Frequency Scale (DDFS, Giambra, 1993) is one of the 28 scales composing the Imaginal Process Inventory (Singer & Antrobus, 1963, 1970) and consists of 12 items assessing daydreaming frequency. Participants are asked to choose on a five-point Likert scale the nature of thoughts which is appropriate for them. For example, “I lose myself in active daydreaming” can be answered by either (1) *Infrequently*, (2) *Once a week*, (3) *Once a day*, (4) *A few times during the day* or (5) *Many different times during the day*. The original 12 items of the DDFS were translated into French by (Stawarczyk, Majerus, Van der Linden, & D’Argembeau, 2012) with a back-translation procedure. Cronbach’s alpha have been computed on the English (α = .92) and French version (α = .92) of the questionnaire and revealed good reliability.

#### Mindfulness

The Mindful Attention Awareness Scale (MAAS, Brown & Ryan, 2003) is a 15-item scale introduced by the following sentences: “*Below is a collection of statements about your everyday experience. Please answer according to what really reflects your experience rather than what you think your experience should be*.” Participants must rate the statements on a six-point Likert scale ranging from 1 (*almost always*) to 6 (*almost never*). For example, “I rush through activities without being really attentive to them” or “I snack without being aware that I'm eating.” A higher score indicates more mindful abilities. The French version of the MAAS was translated by Stawarczyk et al. (2012) with a back-translation procedure. Cronbach’s alpha have been computed on the English (α = .88) and French version (α = .82) of the questionnaire and revealed good reliability.

#### Mood

The Positive and Negative Affect Schedule (PANAS; Watson, Clark, & Tellegen, 1988) consists of two 10-items mood scales that respectively measure positive and negative affect, both as states and traits. Participants are asked to rate the extent to which they experience particular emotions *right now* with reference to a five-point Likert-scale ranging from 1 (*very slightly or not at all*) to 5 (*very much*). Examples of emotions would be as such, “distressed” or “excited.” The French version of the PANAS was translated by Gaudreau, Sanchez, and Blondin (2006) with a back-translation procedure. Cronbach’s alpha have been computed on the English (Positive affect, α = .90; Negative affect, α = .93) and French version (Positive affect, α = .85; Negative affect, α = .91) of the positive and negative subscales of the questionnaire and revealed good reliability.

#### Future Thinking

The Future-Self Thoughts questionnaire (FST; McElwee & Haugh, 2010) consists of 11 items designed to assess two dimensions of thoughts about one’s future selves: (i) Frequency, the extent to which participants spontaneously think of themselves in the future, and (ii) Clarity, the vividness with which participants “see” themselves in the future. Participants are asked to rate the extent to which each statement describes how they think or act in their daily life with reference to a six-point Likert-scale ranging from 1 (*not at all true for me*) to 6 (*completely true for me*). For example, a question like “When I daydream, I often see myself as I may be in the future” measures Future-self thoughts frequency, and a question like “My future seems vague and uncertain to me” measures their clarity. The French version of the FST was translated by Stawarczyk et al. (2012) with a back-translation procedure. Cronbach’s alpha have been computed on the English (Frequency, α = .91; Clarity, α = .58) and French version (Frequency, α = .82; Clarity, α = .57) of the Frequency and Clarity subscales of the questionnaire and revealed good reliability considering the number of items within each subscales (Frequency, 6 items; Clarity, 5 items).

#### Self-Attentiveness

The Rumination and Reflection Questionnaire (RRQ; Trapnell & Campbell, 1999) consists of 24 items designed to assess reflection and rumination where participants are asked to rate the extent to which they agree or disagree to statements. Answers are given on a five-point Likert-scale ranging from 1 (*Strongly disagree*) to 5 (*Strongly agree*). According to Trapnell and Campbell (1999), rumination and reflection both involve heightened attention to self. *Rumination* is "self-attentiveness motivated by perceived threats, losses, or injustices to the self"; *reflection* is "self-attentiveness motivated by curiosity or epistemic interest in the self" (Trapnell & Campbell, 1999, p. 297). For example, “My attention is often focused on aspects of myself I wish I'd stop thinking about” for rumination and “I'm very self-inquisitive by nature” for reflection. This questionnaire was translated to French by Fluckiger (2009). Cronbach’s alpha have been computed on the English (Reflection, α = .86; Rumination, α = .91) and French version (Reflection, α = .82; Rumination, α = .87) of the Reflection and Rumination subscales of the questionnaire and revealed good reliability.

#### Cognitive Failures

The Cognitive Failure Questionnaire (CFQ; Broadbent, Cooper, FitzGerald, & Parkes, 1982) consists of 25 items measuring self-reported failures in perception, memory, and motor function. Participants are told that the questions will be about minor mistakes, which everyone makes from time to time, but some of which happen more often than others. They must rate how often these things have happened to them in the last 6 months on a five-points Likert-scale from 0 (*never*) to 4 (*very often*). For example, “Do you fail to notice signposts on the road?.” The French version used in the present study was translated by Fluckiger (2009). Cronbach’s alpha have been computed on the English (α = .91) and French version (α = .87) of the questionnaire and revealed good reliability.

#### Depressive Symptoms

The Beck Depression Inventory (BDI-II; Beck, Steer, Ball, & Ranieri, 1996) is a widely used tool for assessing the severity of depressive symptomatology. The 21 items are rated on a four-point Likert scale, from 0 to 3 points. Scores range from 0 to 63. For example, “Past failure” can be answered by either (0) *I do not feel like a failure*, (1) *I have failed more than I should have*, (2) *As I look back, I see a lot of failures* or (3) *I feel I am a total failure as a person*. The French version used in this study was done by Fluckiger (2009) and validated in both clinical (depressed) and nonclinical samples. Cronbach’s alpha have been computed on the English (α = .94) and French version (α = .88) of the questionnaire and revealed good reliability.

### Procedure

Recruitment of the participants for this study was via social media and a poster campaign both in France and the United Kingdom (UK). They all took part in this study online using Qualtrics’ survey software (qualtrics.com). All the questionnaires (DDFS, MAAS, PANAS, FST, RRQ, CFQ and the BDI), were presented in a randomized order. Before the end of the session participants had to give demographic information – gender, age, number of years of education, native language and country of residence. Participants took approximately 30 minutes to complete the questionnaires. The survey was completed anonymously, and no compensation was given for participation.

## Results

Pearson’s correlations were initially conducted between each measure for both language conditions (English, French). Analyses replicated previous findings by revealing the commonly found negative correlation linking mind-wandering to age, and positive correlations linking mind-wandering to mindfulness, cognitive failures and depression scores (see Table 1).

**Table 1:** Correlations between Variables for Both English and French Speakers Individually and Jointly

Variable	Native language	1	2	3	4	5	6	7	8	9	10
1. Age	English	-									
	French	-									
	**Total**	-									
2. DDFS	English	-.26***	-								
	French	-.35***	-								
	**Total**	-.31***	-								
3. MAAS	English	.16*	-.49***	-							
	French	.10	-.45**	-							
	**Total**	.13*	-.47***	-							
4. PANAS Positive	English	.11	-.12	-.00	-						
	French	.02	-.13	.19*	-						
	**Total**	.07	-.11	.10	-						
5. PANAS Negative	English	-.08	.24**	-.36***	-.02	-					
	French	-.04	.19*	-.37***	-.09	-					
	**Total**	-.06	.22***	-.35***	.04	-					
6. FST Clarity	English	.03	-.06	.24**	.33***	-.34***	-				
	French	.06	-.27***	.27***	.26***	-.27***	-				
	**Total**	.04	-.17**	.25***	.27***	-.31***	-				
7. FST Frequency	English	-.38***	.35***	-.33***	.08	.15	.13	-			
	French	-.31***	.37***	-.37***	-.05	.22**	.09	-			
	**Total**	-.35***	.36***	-.35***	.02	.18***	.10	-			
8. RRQ Rumination	English	-.31***	.51***	-.47***	-.28***	.41***	-.18*	.32***	-		
	French	-.12	.36***	-.40***	-.27***	.47***	-.23**	.42***	-		
	**Total**	-.22***	.41***	-.44***	-.31***	.42***	-.19***	.36***	-		
9. RRQ Reflection	English	.11	.21***	-.16	.20*	.13	.09	.09	.13	-	
	French	-.06	.27***	-.07	.13	-.04	.10	.24**	.06	-	
	**Total**	.03	.24***	-.09*	.21***	.06	.08	.16***	.04	-	
10. CFQ	English	-.07	.42***	-.74***	-.10	.39***	-.26***	.19 *	.45***	.06	-
	French	-.15	.51***	-.68***	-.24**	.37***	-.25**	.34***	.39***	.09	-
	**Total**	-.11	.46***	-.71***	-.18**	.37***	-.24***	.26***	.43***	.05	-
11. BDI	English	-.13	.39***	-.53***	-.33***	.64***	-.39***	.22**	.58***	.11	.58***
	French	-.20*	.43***	-.38***	-.53***	.54***	-.37***	.28**	.55***	-.08	.47***
	**Total**	-.16**	.40***	-.46***	-.40***	.59***	-.38***	.24***	.54***	.03	.53***

*Note*. DDFS = Daydreaming Frequency Scale; MAAS = Mindful Attention Awareness Scale; PANAS = Positive and Negative Affect Schedule; FST = Future-Self Thoughts; RRQ = Rumination and Reflection Questionnaire; CFQ = Cognitive Failure Questionnaire; BDI = Beck Depression Inventory.

*p < .05. **p < .01. ***p < .001.

### Effect of Native Language

In order to investigate the differences between French and English native speakers on the subsequent Analyses of Variance (ANOVA) have been conducted on participants’ scores to the questionnaires (DDFS, MAAS, PA, NA, FST Clarity, FST Frequency, Rumination, Reflection, CFQ, and BDI). Regarding positive mood, an effect of native Language indicate that English speakers tended to have less positive thoughts than French speakers, *F*(1, 306) = 15.22, *p* < .001, $\eta_{p}^{2}$ = .05. Considering rumination, results showed an effect of native Language, indicating that English speakers tended to ruminate more than French speakers, *F*(1, 306) = 14.70, *p* < .001, $\eta_{p}^{2}$ = .05. Finally, results on the reflection measure revealed an effect of native Language, indicating that English speakers tended to reflect less than French speakers, *F*(1, 306) = 19.70, *p* < .001, $\eta_{p}^{2}$ = .06. Mean and standard deviation scores for each measure are presented in Table 2. In summary, results demonstrated that French native speakers reported more positive emotions, ruminated less and experienced more reflection than English native speakers.

**Table 2:** Mean Scores, Standard Deviation and Number of Participants of Pertinent Measures

Measure	English Speakers			French Speakers		
	*M*	*SD*	*N*	*M*	*SD*	*N*
DDFS	3.46	.74	154	3.53	0.83	154
MAAS	3.98	.87	154	4.11	0.77	154
PANAS – Positive***	2.57	.74	154	2.90	0.73	154
PANAS – Negative	1.79	.79	154	1.91	0.83	154
FST – Clarity	3. 61	1.11	154	3.46	1.20	154
FST – Frequency	3.65	1.31	154	3.63	1.16	154
RRQ – Rumination***	3.73	.85	154	3.36	0.87	154
RRQ – Reflection***	3.27	.72	154	3.65	0.78	154
CFQ	1.84	.60	154	1.74	0.62	154
BDI	15.76	12.43	154	16.04	9.78	154

*Note.* DDFS = Daydreaming Frequency Scale; MAAS = Mindful Attention Awareness Scale; PANAS = Positive and Negative Affect Schedule; FST = Future-Self Thoughts; RRQ = Rumination and Reflection Questionnaire; CFQ = Cognitive Failure Questionnaire; BDI = Beck Depression Inventory.

***p < .001.

### Effect of Age and Native Language

In order to investigate individual changes across the life span, both samples were divided into three age groups (Table 3; Young: 18–35, Middle-aged: 36–54 and Older adults: 55+).

**Table 3:** Age Means, Standard Deviations and Number of Participants for Each Defined Age Groups for Both the English and French Native Speaker Samples

Age groups	English			French			Total		
	*M*	*SD*	*N*	*M*	*SD*	*N*	*M*	*SD*	*N*
Young	21.77	4.36	108	22.55	3.92	105	22.15	4.16	212
Middle-age	44.40	4.71	25	46.03	5.72	31	45.30	5.31	56
Old	64.10	5.79	21	65.72	5.72	18	64.85	5.48	39
Total	31.21	16.15	154	32.32	15.96	154	31.77	16.04	308

#### Mind-Wandering

A 3 (Age; *Young, Middle-Aged, Old*) by 2 (Language; *English, French*) ANOVA was conducted on the DDFS daydreaming scores. A main effect of Age was significant, *F*(2, 302) = 12.63, *p* < .001, $\eta_{p}^{2}$ = .08, indicating that Young adults (*M* = 3.62, *SD* = 0.73) and Middle-aged adults (*M* = 3.38, *SD* = 0.81) tend to mind wander more frequently and Older adults (*M* = 2.97, *SD* = 0.82), *p* < .001, *p* = .022. Young and Middle-aged adults did not differ in their mind-wandering scores, *p* = .159.

**Figure 1:**
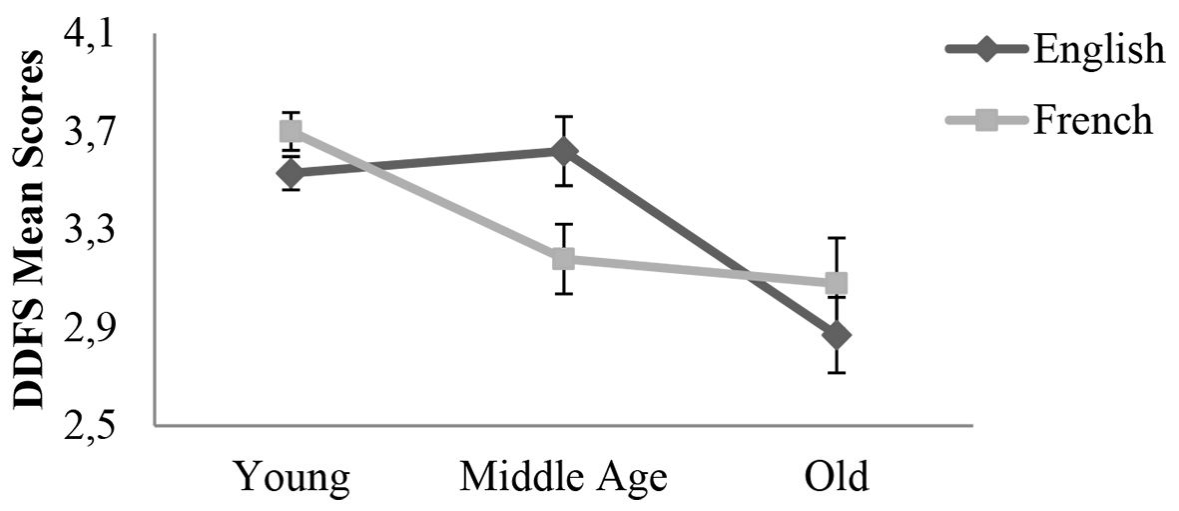
The effects of participants’ native Language (*English*, *French*) and their age group (*Young*, *Middle age*, *Old*) on mind-wandering frequency measured by the DDFS. *Note.* DDFS = Daydreaming Frequency Scale. Error bars represent Standard Errors.

The interaction between Language and Age was significant, *F*(2, 302) = 3.93, *p* = .021, $\eta_{p}^{2}$ = .03. The decomposition of this interaction revealed that in the English speaker group, the effect of Age was significant, *F*(2, 151) = 8.69, *p* < .001, $\eta_{p}^{2}$ = .10, indicating that Young (*M* = 3.53, *SD* = 0.73) and Middle-aged adults (*M* = 3.62, *SD* = 0.54) were not different in their mind-wandering frequency. However, both Young and Middle-aged adults reported more mind-wandering than Older adults (*M* = 2.87, *SD* = 0.76), respectively *p* < .001 and *p* = .001. In the French speaker group, the effect of Age was also significant, *F*(2, 151) = 8.44, *p* < .001, $\eta_{p}^{2}$ = .10. Results showed that Young adults (*M* = 3.70, *SD* = 0.73) had higher mind-wandering frequency than Middle-aged (*M* = 3.18, *SD* = 0.93), *p* = .004 and Older adults, (*M* = 3.09, *SD* = 0.89), *p* = .008. However, Middle-aged adults and Older adults mind-wandering frequency was equivalent, *p* = .999. These data presented in Figure 1 demonstrate an earlier decrease in mind-wandering frequency in the French speaking group (between 36 and 54 years old).

#### Mindfulness

A 3 (Age; *Young, Middle-Aged, Old*) by 2 (Language; *English, French*) ANOVA was conducted on MAAS scores. A main effect of Age was evidenced, *F*(2, 302) = 3.57, *p* = .029, $\eta_{p}^{2}$ = .02, indicating that Young (*M* = 4.00, *SD* = 0.82) had a tendency to be less mindful than Older adults (*M* = 4.37, *SD* = 0.80), *p* = .033. Young and older adults did not differ from Middle-aged adults (*M* = 3.98, *SD* = 0.81) on this score, respectively *p* = .999 and *p* = .058.

Results suggested an increase of mindfulness as one grows older, in line with results regarding mind-wandering. However, mindfulness does not seem to be influenced by cultural differences between the two populations.

#### Mood

A 2 (Valance; *Positive*, *Negative*) by 3 (Age; *Young*, *Middle*-*Aged*, *Old*) by 2 (Language; *English*, *French*) ANOVA was conduct on PANAS scores. The main effect of Valence was significant, indicating that participants tended to express more positive affect (*M* = 2.74, *SD* = 0.75) than negative (*M* = 1.85, *SD* = 0.81), *F*(1, 302) = 131.09, *p* < .001, $\eta_{p}^{2}$ = .30. The main effect of native Language revealed that English participants (*M* = 2.18, *SD* = 0.54) tend to express less affect than their French counterparts (*M* = 2.40, *SD* = 0.53), *F*(1, 302) = 7.21, *p* = .008, $\eta_{p}^{2}$ = .02.

Results revealed a general tendency for participants to express more positive affect than negative ones. In addition, French native speakers tended to be more expressive than English native speaker.

#### Future Thinking

A 2 (Specificity; *Clarity, Frequency*) by 3 (Age; *Young, Middle-Aged, Old*) by 2 (Language; *English, French*) repeated measure ANOVA was conduct on FST scores. The main effect of Age was significant, *F*(2, 302) = 6.63, *p* = .002, $\eta_{p}^{2}$ = .04, indicating that Young adults (*M* = 3.58, *SD* = 0.75), scored higher on the Future-self thoughts questionnaire than Older adults (*M* = 2.96, *SD* = 0.78), *p* = .002. Young and Older adults did not differ from Middle-aged adults (*M* = 3.23, *SD* = 1.01) on these scores, respectively, *p* = .231 and *p* = 336.

**Figure 2:**
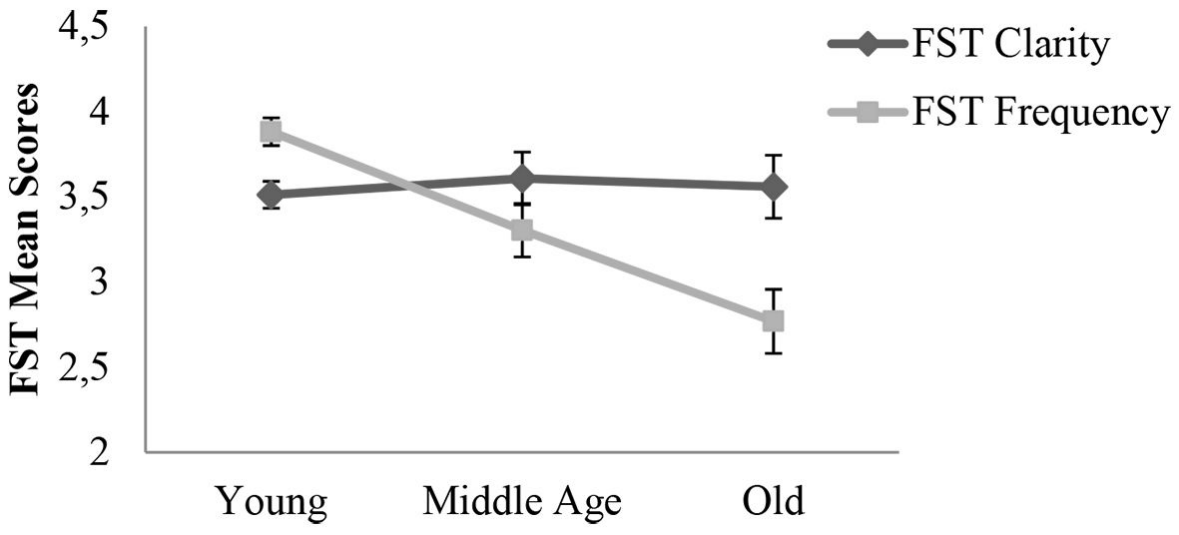
The effect of specificity of future-self thought (*Clarity*, *Frequency*) and participants’ age group (*Young*, *Middle-age*, *Old*) on the measure of future-self thoughts (FST). *Note.* Error bars represent Standard Errors.

The interaction between Specificity and Age was significant, *F*(2, 302) = 11.01, *p* < .001, $\eta_{p}^{2}$ = .07 (Figure 2). The decomposition of the interaction showed that Future-self thoughts clarity did not change across life span, *F*(2, 305) = .163, *p* = .850, $\eta_{p}^{2}$ = .00, whereas its frequency did, *F*(2, 305) = 17.495, *p* < .001, $\eta_{p}^{2}$ = .10. Results revealed that Young adults (*M* = 3.88, *SD* = 1.17) had more frequent Future-self thoughts than Middle aged adults (*M* = 3.30, *SD* = 1.31), *p* = .004 and Older adults (*M* = 2.77, *SD* = 0.98), *p* < .001. Middle-aged adults and Older adults had a similar frequency of Future-self thoughts, *p* = .090.

Therefore, results demonstrate that the frequency of future-self thoughts decreases with age for both French and English speakers. The clarity of the thoughts was, however, not influenced by either age or culture.

#### Self-Attentiveness

A 2 (Self-Attentiveness; *Rumination, Reflection*) by 3 (Age; *Young, Middle-Aged, Old*) by 2 (Language; *English, French*) repeated measures ANOVA was conduct on RRQ scores. The main effect of Age was significant, indicating that Older adults (*M* = 3.27, *SD* = 0.59) had less self-attentive thoughts Young adults (*M* = 3.55, *SD* = 0.59), *p* = .031, *F*(2, 302) = 3.35, *p* = .037, $\eta_{p}^{2}$ = .02. Young and Older adults did not differ from Middle-aged adults (*M* = 3.49, *SD* = 0.61) on self-attentiveness, respectively *p* = .999 and *p* = .218. The three-way interaction between Self-Attentiveness, Language and Age was not significant, *F*(2, 302) = 1.20, *p* = .303, $\eta_{p}^{2}$ = .01, however, the three two-way interactions revealed interesting findings.

The interaction between Self-Attentiveness and Age was significant, *F*(2, 302) = 4.829, *p* = .009, $\eta_{p}^{2}$ = .03. Further analyses revealed that Reflection rates did not change across life span, *F*(2, 305) = 1.01, *p* = .365, $\eta_{p}^{2}$ = .01, whereas Rumination rates did, *F*(2, 450) = 7.36, *p* = .001, $\eta_{p}^{2}$ = .03. As such, Young adults (*M* = 3.66, *SD* = 0.06) ruminated more than Older adults (*M* = 3.16, *SD* = 0.14), *p* = .003. Middle-aged (*M* = 3.39, *SD* = 0.12) adults did not differ from young or older adults on this measure, respectively *p* = .112 and *p* = .607.

The interaction between Self-Attentiveness and native Language was significant *F*(1, 302) = 8.33, *p* < .001, $\eta_{p}^{2}$ = .05. Further analyses showed that both rumination and reflection rates differed as a condition of culture, respectively *F*(1, 306) = 14.70, *p* < .001, $\eta_{p}^{2}$ = .05 and *F*(1, 306) = 19.70, *p* < .001, $\eta_{p}^{2}$ = .06. However, English native speaker presented higher rates of rumination (*M* = 3.73, *SD* = 0.85) and lower rates of reflection (*M* = 3.27, *SD* = 0.72) than French native speakers (Rumination, *M* = 3.36, *SD* = 0.87; Reflection, *M* = 3.65, *SD* = 0.78).

The interaction between Language and Age was significant, *F*(2, 302) = 3.99, *p* = .018, $\eta_{p}^{2}$ = .03. Decomposition of the interaction demonstrated that in the case of French native speakers Age did not have an impact on the overall measure of Self-Attentiveness, *F*(2, 151) = 1.81, *p* = .168, $\eta_{p}^{2}$ = .02. However, Age seemed to have an impact on the overall measure of self-attentiveness in the English native speaker’s group, *F*(2, 151) = 6.06, *p* = .003, $\eta_{p}^{2}$ = .07. As such, Young (*M* = 3.54, *SD* = 0.06) and Middle-aged adults (*M* = 3.68, *SD* = 0.12) presented higher rates of self-attentiveness than older adults (*M* = 3.12, *SD* = 0.13), respectively *p* = .008 and *p* = .004. Young and Middle-aged adults presented similar scores on this measure, *p* = .810.

**Figure 3:**
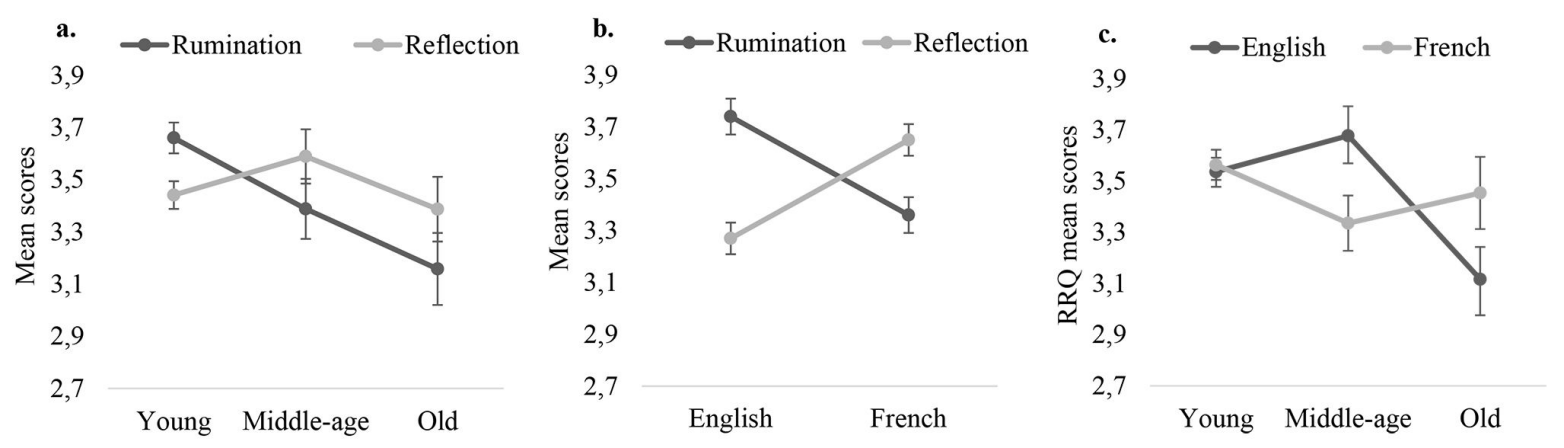
a. The effect of participants’ age (*Young*, *Middle age*, *Old*) on Rumination and Reflection rates. b. The effect of participants’ native Language (*English*, *French*) on Rumination and Reflection. c. The effect of participants’ native Language (*English, French*) and their age group (*Young, Middle age, Old*) on their overall self-attentiveness rates. *Note*. RRQ = Rumination and Reflection Questionnaire. Error bars represent Standard Errors.

Results demonstrated cultural and age difference regarding attention toward the self as measured by rumination and reflection. Firstly, findings showed that only Rumination rates seems to be impacted by age with a decrease of such thoughts as age increases. Secondly, French and British citizens presented an opposite pattern of self-attentiveness with French native speakers engaging more in self-reflection and less in rumination than English native speakers. Finally, self-attentiveness revealed to be stable across time in the French population but not the British one, where a decrease occurs in later age. These data are summarised in Figure 3.

#### Cognitive Failures

A 3 (Age; *Young, Middle-Aged, Old*) by 2 (Language; *English, French*) ANOVA was carried out on the CFQ scores and revealed no significant effects. Surprisingly, findings did not evidence any age effect on this measure of cognitive failure.

#### Depressive Symptoms

A 3 (Age; *Young, Middle-Aged, Old*) by 2 (Language; *English, French*) ANOVA was carried out on BDI scores. A significant main effect of Age was evidenced, *F*(2, 302) = 4.47, *p* = .012, $\eta_{p}^{2}$ = .03, indicating that Young adults (*M* = 16.83, *SD* = 11.58) tended to score higher on the depression inventory than Older adults (*M* = 10.95, *SD* = 9.70), *p* = .009. Middle-aged adults (*M* = 15.80, *SD* = 9.69) scores on the depression inventory were similar to both Young, *p* = .999 and Older adults, *p* = .090.

Results regarding depressive symptoms did not reveal any cultural differences but did show an effect of age, indicating that young adults tended to be more depressed than older adults.

### Confirmatory Factor Analysis

Based on the literature and the abovementioned primary analyses it is clear that mind-wandering experiences are eclectic by nature. Specifically, three features seem to be associated to mind-wandering experiences, namely self-orientation, negative mood and attentional skills. In this study, the self-related construct was measured by the reflection and future-self thoughts frequency subscales; the negative construct was measured by the depressive symptoms, negative affect and rumination scales; and finally, the attentional construct was measured by the mindfulness and cognitive failures questionnaires. In consideration of the literature, the model will also implement age as a factor influencing mind-wandering frequency. To assess formally the fit of our measurement model to the data, we used confirmatory factor analysis (CFA). Conclusions about structural model fits were based on commonly used fit indices with cut-offs suggested by Kline (2005): χ^2^/*df* < 2; CFI > 0.90 for reasonably good fit; RMSEA < 0.05 for good fit, < 0.08 for reasonable fit.

**Figure 4:**
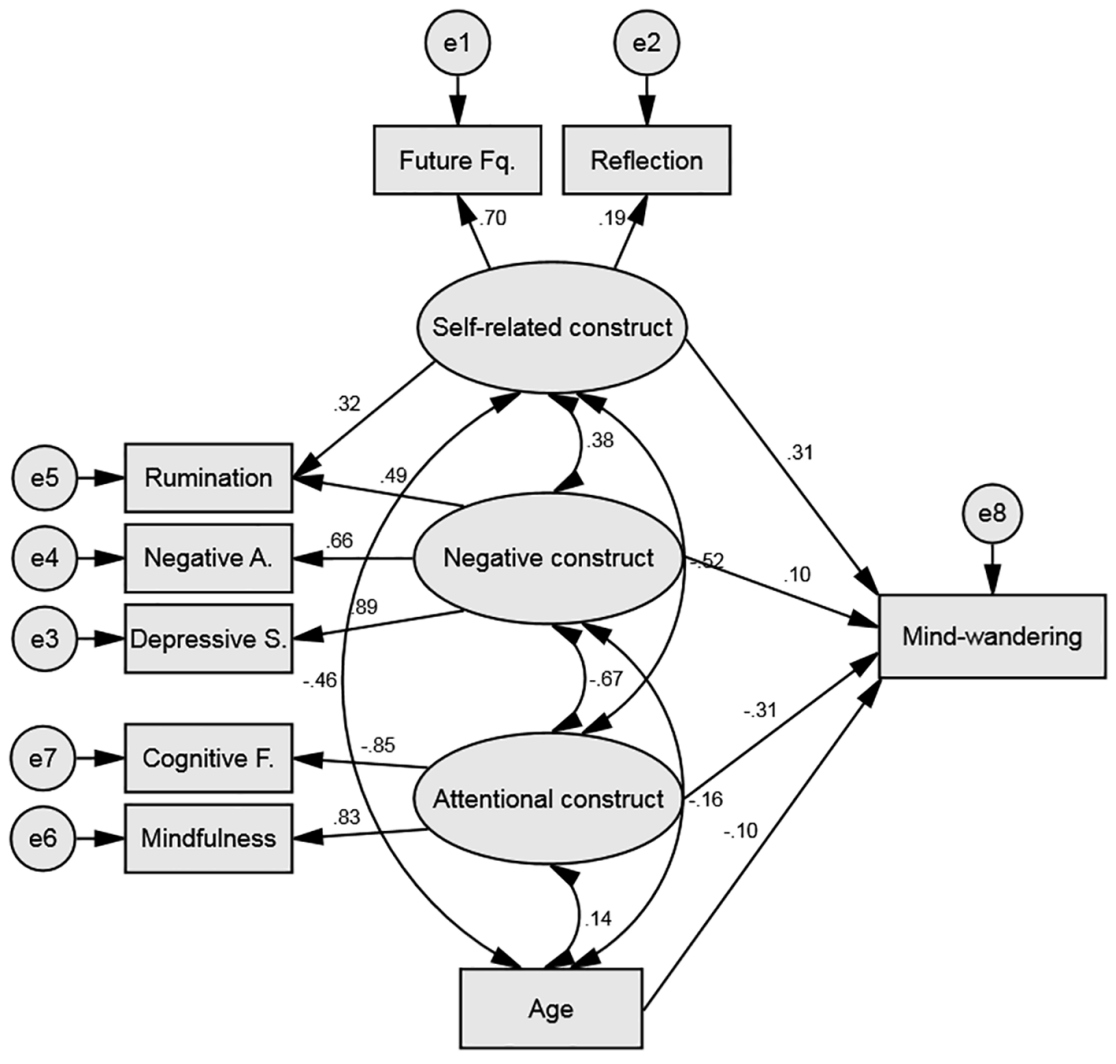
Structural equation model predicting mind-wandering frequency with self-related, negative and attentional constructs and age. *Note*. Single-headed arrows connecting latent variables (circles) and observed variable (rectangles) represent standardized path coefficients, indicating the contribution of the variables. Double-headed arrows connecting the factors represent the correlations among the factors.

The fit indices of the model were as follows; χ^2^(19) = 60.80, *p* < .001, χ^2^/*df* = 3.20, CFI = 0.949, RMSEA = 0.085. All measures loaded significantly on the negative and attentional construct. Reflection marginally loaded on the self-related construct (.189). However, it seemed that only Age (-.155) and the Attentional construct (-.363) significantly predicted mind-wandering. Each of the four factors (self-related, negative, attentional and age) co-varied significantly with each other. Although we suspected rumination to be largely associated with the Negative Construct, by definition rumination has a self-related component. Therefore, with the aim of improving our model, rumination scores were added to the self-related construct. This change was based on modification indexes from this analysis, and in accordance with the theory since definition ruminative thoughts are self-related. This new model, presented in Figure 4, showed to have a reasonably good fit; χ^2^(18) = 40.29, *p* = .002, χ^2^/*df* = 2.24, CFI = 0.973, RMSEA = 0.064. As can be seen the fit of the model was improved and, this time, each measure loaded significantly on its construct of interest. In this new model only the attentional (-.308) and self-related constructs (.314) predicted mind-wandering frequency and the four factors co-varied significantly with each other.

Next, a group comparison was conducted to identify if the fit of the model differed between French and English native speakers. To do so two models where compared, one allowing for variability between the two groups (H_1_) and one restricting the loading of the 4 factors (self-related, negative, attentional and age) to an equal number in both populations (null hypothesis, H_0_). Although the overall fit statistics show that both models are a good fit, H_1_, χ^2^(36) = 58.86, *p* = .009, χ^2^/*df* = 1.63, CFI = 0.973, RMSEA = 0.046; H_0,_ χ^2^(40) = 73.23, *p* = .001, χ^2^/*df* = 1.83, CFI = 0.961, RMSEA = 0.052, the nested model comparison that assesses the worsening of overall fit due to imposing the four restrictions on the original model shows to be statistically significant χ^2^(4) = 14.37, *p* = .006. This finding suggests that the two models differ and that the model with equal factor loadings (H_0_) should be rejected in favour of the original model (H_1_). Consequently, the model created here does not fit French and English native speakers equally and therefore separation is warranted.

**Figure 5:**
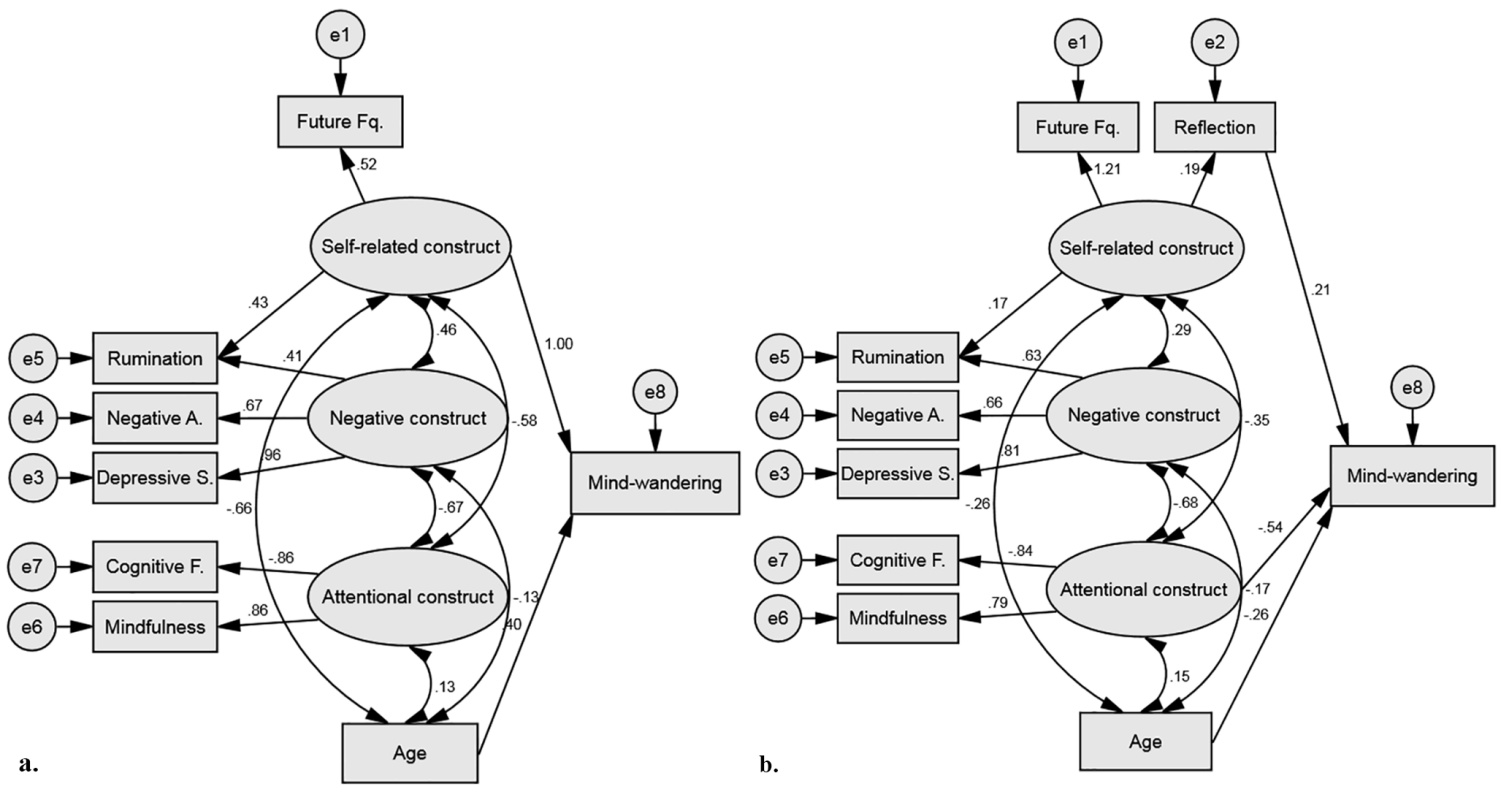
a. Structural equation model predicting mind-wandering frequency with self-related construct and age in a population of native English speakers. b. Structural equation model predicting mind-wandering frequency with reflection scores, attentional construct and age in a population of native French speakers. *Note*. Single-headed arrows connecting latent variables (circles) and observed variable (rectangles) represent standardized path coefficients, indicating the contribution of the variables. Double-headed arrows connecting the factors represent the correlations among the factors

Looking only at *English native speakers* the model showed to have a reasonably good fit; χ^2^(18) = 27.17, *p* = .076, χ^2^/*df* = 1.51, CFI = 0.980, RMSEA = 0.058. As shown in Table 4 the negative (.030) and attentional constructs (-.001) did not significantly contributed to mind-wandering frequency. Therefore, the model was improved by removing these two direct effects. The fit of the new model was good; χ^2^(20) = 27.19, *p* = .130, χ^2^/*df* = 1.36, CFI = 0.984, RMSEA = 0.048. However, the contribution of reflection was not significant (.118). Therefore, with the aim to improve further this model the measure of reflection was removed. The third model created for the English native speakers was of very good fit; χ^2^(13) = 12.17, *p* = .514, χ^2^/*df* = 0.94, CFI = 1.00, RMSEA = 0.000. This final model is presented in Figure 5a. As can be seen, in this population only age and self-related thoughts are directly influencing mind-wandering frequency. It is worth noting that the negative (.457) and attentional constructs (-.582) significantly co-varied with the self-related construct and most likely contributed indirectly to mind-wandering frequency.

**Table 4:** Factor Loading for Models with Data from Both Groups Separately

Factor	English native speakers			French native speakers		
	Model 1	Model 2	Model 3 (Figure 5a)	Model 1	Model 2	Model 3 (Figure 5b)
Regression						
Self-related						
Reflection	.117	.118	-	.233	.211	.195
Future Fq.	.528^a^	.525^a^	.523^a^	1.026^a^	1.122^a^	1.209^a^
Rumination	.446***	.448***	.434***	.208	.184	.166
Negative						
Depressive S.	.959^a^	.958^a^	.955^a^	.819^a^	.810^a^	.811^a^
Negative A.	.667***	.667***	.669***	.659***	.665***	.664***
Rumination	.401***	.392***	.406***	.609***	.626***	.635***
Attentional						
Cognitive F.	-.860***	-.860***	-.861***	-.855***	-.845***	-.844***
Mindfulness	.863^a^	.862^a^	.861^a^	.795^a^	.794^a^	.794^a^
Mind-wandering						
Self-related	.933***	.963***	1.00***	.068	-	-
Negative	.030	-	-	.102	-	-
Attentional	-.001	-	-	-.456***	-.562***	-.545***
Age	.323	.338*	.403*	-.245***	-.267***	-.258***
Reflection	-	-	-	-	-	.209***
Covariance						
Self-related						
Negative	.449**	.467***	.457***	.330***	.307***	.288***
Attentional	-.592***	-.598***	-.582***	-.410***	-.385***	-.352***
Age	-.625***	-.626***	-.664***	-.300***	-.276***	-.257***
Negative						
Attentional	-.669***	-.670***	-.672***	-.649***	-.668***	-.677***
Age	-.132	-.132	-.132	-.169^†^	-.170^†^	-.173^†^
Attentional						
Age	.134	.134	.134	.153	.153	.153

^a^regression weighed at 1.

^†^*p* < .07. *p < .05. **p < .01. ***p < .001.

Looking only at French native speakers the model revealed to have a reasonable fit; χ^2^(18) = 31.69, *p* = .024, χ^2^/*df* = 1.76, CFI = 0.965, RMSEA = 0.071. As shown in Table 4 the self-related (.068) and negative constructs (.102) did not significantly contributed to mind-wandering frequency. Therefore, the model was improved by removing these two direct effects. The fit of the new model was reasonably good; χ^2^(20) = 33.23, *p* = .032, χ^2^/*df* = 1.66, CFI = 0.967, RMSEA = 0.066. Based on modification indexes from this analysis this model was further improved by added a direct path between reflection and mind wandering frequency. The third model created for the French native speakers was of good fit; χ^2^(19) = 22.29, *p* = .270, χ^2^/*df* = 1.17, CFI = 0.992, RMSEA = 0.034. This final model is presented in Figure 5b. As can be seen, in this population it is age, attentional skills and reflection that are directly influencing mind-wandering frequency. It is worth noting that the self-related (-.352) and negative constructs (-.677) significantly co-varied with the attentional construct and most likely contributed indirectly to mind-wandering frequency. Finally, rumination scores did not significantly loaded into the self-related construct (.166), yet removing it from this construct did not improve the model fit; χ^2^(20) = 30.51, *p* = .062, χ^2^/*df* = 1.53, CFI = 0.973, RMSEA = 0.059.

## Discussion

Given the myriad evidence demonstrating strong socio-cultural influences on cognition and behaviour, it is surprising that cultural impacts on internal trains of thought have been neglected. This study aimed to investigate the influence of culture on mind-wandering experience across different age groups, by comparing a population of English (UK) and French (France) speakers. Our approach considered not only differences in the frequency of mind-wandering but critical characteristics and content that shape the occurrence of such self-generate thoughts. Overall, the data highlighted how culture drives differences in age-related declines in mind-wandering frequency. Importantly, socio-cultural experience impacted on the frequency of relatively more negative (rumination) and positive (reflection) thoughts, as well as participants’ overall affective expressiveness. Finally, evidence suggest that different theoretical models, for the French and British population, capture the complex relationships between mind-wandering and related features.

Consider first the frequently observed age-related decrease in mind-wandering frequency. Research using both self-report questionnaires (Giambra, 1989) and experience sampling in laboratory settings (Jackson & Balota, 2012) has demonstrated a reduction of mind-wandering as we age. Early explanations focussed on the cognitive capacity view of ageing to explain this counter intuitive finding. As we grow older our capacity to direct attention and resources to the task at hand declines and as a result older adult have less ability to switch or share resources between activities and internal trains of thought. The executive failure account of mind-wandering experience (Riby, Smallwood, & Gunn, 2008; Smallwood & Schooler, 2015) suggests that one’s cognitive control enables the flexible adjustment of mind-wandering events, such as reducing its occurrence in the context of a difficult task. In a similar vein, older adults’ lower cognitive control may prevent them from engaging in mind-wandering experience while performing cognitive tasks. Co-ordinating multiple tasks and executive deficits of this nature have been widely reported in the ageing literature (for meta-analysis see Riby et al., 2004a). Interestingly, during ongoing tasks if we probe both task-related and task-unrelated thoughts, older adults tend to report concerns about their performance. This task-related interference has been reported to be more dominant in older adults than young adults (e.g., McVay, Meier, Touron, & Kane, 2013). Anxiety, worry and monitoring of performance for older adults may demand cognitive resources to maintain performance and further reduce the possibility of task-unrelated thoughts to emerge. When comparing the two populations, results evidenced an earlier decrease in mind wandering for the French speakers (36–54 years old) than for the English speakers (over 55 years old). Most research in ageing does not include this middle age group in their investigation; yet findings suggest that this period may well be subject to changes in mind-wandering rate. Early stage theories (Erikson, 1978) suggest moving from middle-age to later adulthood is a critical point in development and many moderators of successful progression and ageing have been identified. Sociocultural context impacts on the roles, beliefs, values and shape our identity and thought processes even when we are approaching later adulthood. Together, this suggests that individuals’ sensitivity to changes at this time of life can support modifications in mind-wandering experiences. However, one must consider the nature of the items composing the DDFS. The focus is mainly made on one’s general tendency to mind wander as a whole which perhaps is not sensitive enough to capture the very nature of the different thought processes that are included under the umbrella terms daydreaming and mind-wandering. As such, questionnaires measuring more specific type of thoughts can be highly informative.

Our examination of individual difference in self-focus (rumination vs. self-reflection) is particularly informative regarding the abovementioned age-related declines in mind-wandering frequency. While reflection rates seem stable across ages, rumination rates evidenced a drop in later adulthood. Rumination involves negatively toned thought content (Nolen-Hoeksema et al., 2008). Moreover, rumination has been associated with perceived threats, losses or injustice to the self (Joireman, 2004) and the daydreaming style *Guilt-fear of Failure* (characterised by anguished fears, fantasies and aggression; Shrimpton, McGann, & Riby, 2017). During early adulthood, a large amount of decisions made will inevitably shape ones’ future in the short and/or long term. As evidenced by the literature on the reminiscence bump, this period of life is known to be filed with key life events such as falling in love, starting a new job and building a family (Rathbone, Moulin, & Conway, 2008). Inevitably, at the time where such important events are experienced or anticipated, one can expect greater future-related thoughts (i.e., see results on future-self thoughts; Stawarczyk et al., 2012) but also greater anguished fears, fantasies or perceived threats to the self. Critically, these elements largely support ruminative thoughts and could explain the higher rates found in younger adults compared to older adults. This interpretation is further corroborated by younger adults’ tendency to be subject to anxiety and depressive symptoms (Jorm, 2000), which are commonly associated with higher rates of rumination (Nolen-Hoeksema, 2000). Now, in contrast with rumination, self-reflection is defined as self-attentiveness motivated by curiosity in the self. A self-reflective thinking style enables problem solving and promotes good mental health (Takano & Tanno, 2009; Trapnell & Campbell, 1999). Previous research from our laboratory has highlighted the positive nature of such thoughts with reflection associated with the *positive-constructive daydreaming* style (Shrimpton et al., 2017). Here we suggest that the resilient features of self-reflection, such as openness to experience, self-knowledge (Trapnell & Campbell, 1999), perspective taking (Joireman, Parrott, & Hammersla, 2002) and the ability to learn from our mistakes (Joireman, 2004), are essential across all stages of life. Major self-reassessment triggered by social and personal events occur at any moment in life, whereas it is choosing a career path, building a family, managing teenagers, ageing parents or adapting to physical and cognitive changes in later adulthood (Demo, 1992; Helson & Moane, 1987; Neugarten, 1974). Therefore, it is not surprising that the therapeutic value of self-reflection is used across all ages (Appelbaum, 1973; Conte et al., 1990).

Focusing on the impact of culture, rumination rates were found higher for English than French native speakers, whereas the opposite pattern was evidenced for reflective rates. As previously outlined, self-reflection is of great value for openness to experience, self-knowledge, perspective taking and the ability to learn from our mistakes. The benefits of self-reflection are in line with more general positive outcomes associated with mind-wandering including creativity, goal planning and future planning (Koestner, Lekes, Powers, & Chicoine, 2002; Smallwood et al., 2011; Verhaeghen, Aikman, & Joormann, 2014). Therefore, reflection seems particularly important in the context of positive life changes as opposed to the relatively more negative rumination counterpart. Bearing in mind the results on mood (i.e., see discussion below), one could infer that the higher emotional expressiveness, found in French speakers, may enable a larger gathering of self-knowledge throughout life; therefore, raising reflections rates. Interestingly, some features of this population illustrate this idea. For example, rates of professional reconversion elicited in France are relatively high; in 2012, a survey was conducted in France indicating that 60% of the working population declared having changed their area of work at least once (Ipsos, 2012). Interestingly, among those, 55% declared that the change was by their own will and not because of dismissal, and 71% stated that this decision marked a change in their life beyond the professional aspect of things. In this context, great self-reflection skills are required, such as the ability to learn from one’s mistakes, or openness to new experiences. Despite the lack of research on the impact of culture on reflection’ rates, these elements suggest a concrete explanation of the French speakers’ higher rates of reflection. In contrast, British citizens’ propensity to be less expressive about their emotions, can be interpreted as a tendency to avoid the processing of feelings. Rumination is related to more avoidance and extrinsic content of goals (Thomsen, Tønnesvang, Schnieber, & Olesen, 2011), while expressive writing interventions showed to reduce the occurrence of ruminative thoughts in formerly depressed patients (Gortner, Rude, & Pennebaker, 2006). Together, this suggests that British citizens’ tendency to overlook their feelings may foster ruminative thoughts as opposed to reflective ones.

Ultimately, the impact of culture and age on the overall rates of self-focused thoughts are perfectly in line with the previously discussed findings. Self-centred thoughts showed to be stable in the French native speakers’ population, while a significant decreased occurred for English native speakers in later adulthood. Keeping in mind that French citizens self-focused thoughts are largely composed of self-reflection and that self-reflection was evidenced as a stable feature across ages, one may consider that the unchanged rates of self-focus in this population is an expression of self-reflection foremost. Similarly, the observed decrease of self-focus thoughts for older native English speakers may be driven by the decrease of ruminative thoughts in this stage of life and the reliance on these type of thoughts for the British population.

A further critical component influencing mind-wandering considered here is mindfulness abilities across the lifespan. This finding has been evidenced previously in the ageing literature, yet no clear interpretation on why this increase occurs has been suggested (Splevins et al., 2009). Mindfulness is defined as the ability to be focused on the present moment and often involves greater attention to detail and effective processing of stimuli in the environment. This increase in mindfulness may illustrate the age-related decrease of mind-wandering frequency and the increase of performance monitoring (i.e., Task relevant interference; McVay et al., 2013). A somewhat simplistic view is that both concepts have been considered as opposite constructs. However, numerous examples suggested that both mind-wandering and mindfulness can have benefits as both increase creativity (Baird et al., 2012; Lebuda, Zabelina, & Karwowski, 2016). Research should further explore this apparent paradox.

Another component supporting the age-related decrease in mind-wandering frequency is the reported reduction in the frequency of future-self thoughts with increased age. Prior research has demonstrated a decrease in future-self thought frequency as one gets older, with no age differences regarding the clarity of the thoughts (Berntsen et al., 2015; Stawarczyk et al., 2012). That is, the quality and vividness of the thoughts remains stable, merely their quantity decreases. This effect on the frequency may be due to a decrease in future perspectives as we grow old, thus inducing either, less future-selves to think about or, a personal bias that leads to progressively avoid future perspectives, consciously or not. From the literature the important contribution of future thinking to mind-wandering episodes is explicit (Baird et al., 2011; D’Argembeau et al., 2011; Smallwood, Nind, et al., 2009). Therefore, this suggests that the reduced frequency of self-future thoughts experienced by older adults will inevitably induce a reduction of the general mind-wandering frequency.

The present study also incorporated measures of depression as well as rumination, both previously identified as important components of mind wandering. Depressive symptoms are closely associated with rumination (Austin, Mitchell, & Goodwin, 2001; Nolen-Hoeksema, 2000) and related research has reported higher mind-wandering frequency in dysphoric patients (Smallwood et al., 2005). Results here evidenced a decrease of depressive symptoms with age, alongside the decrease of mind-wandering frequency and future thinking. This age-related decrease in depressive symptoms, that may appear counter intuitive, has been illustrated in the literature for a “young old” population. Indeed, previous researches on age differences in depression have frequently shown the “young old” (i.e., 55 to 75; Neugarten, 1974) to be less depressed than middle-aged and younger adults (Gatz & Hurwicz, 1990; Jorm, 2000). However, the “old old” adults tend to have quite high levels of depression. Here, in the older adults' group, participants were above 55 years of age with an average of 64.85, therefore, fitting into the “young old” category. Altogether, findings suggest that the age-related decrease of depressive symptoms could contribute to the age-related reduction of mind-wandering episodes, along with the reduced future thinking frequency and increased mindfulness abilities. Therefore, giving further insight into the relationship between depressive thoughts and mind-wandering.

Nevertheless, another element was theoretically expected to support the age-related decrease in mind-wandering frequency. Mind-wandering has long been associated to attentional deficits, thus indicating the importance to measure cognitive failures. Older adults have widely been reported to have difficulties on measures of executive control (e.g., Braver & West, 2008) but self-reported failures were not observed here. However, other studies seem be in line with our findings. For example, a normative study did not evidence any correlations between CFQ scores (Broadbent et al., 1982) and any demographic factors, including age. This may be explained by a difficulty for older adults to report their cognitive failures or more general difficulties in the sensitivity of the measure to capture change (especially given the data was acquired online) compared to laboratory measures of executive dysfunction. Indeed, older adults tend to claim to have remarkable worries about their own cognitive abilities, but do not seem to be able to record the cognitive lapses that are known to increase in aging (Mecacci & Righi, 2006). Additionally, data acquisition (i.e., online study) is a cofounding factor that needs to be kept in mind when considering this result, as it may have induced a selective bias.

Limited work has been carried out on individual differences in thought processes across French and English speakers. Therefore, precise predictions regarding the impact of emotionality of thought content is difficult to generate. Overall, English speakers showed less extreme positive and negative emotions than their French counterparts. Although investigations of cultural differences in emotional expressiveness are rare, lay observations of beliefs and behaviours of French and British citizens suggest cultural differences in the expression of their emotions. British citizens have an intriguing relationship with their own and other people’s emotions; they tend not to be overly demonstrative, and look down on gushing public displays of emotion (Khor & March, 2007; Smallwood, Fitzgerald, Miles, & Phillips, 2009). This tendency to hide their feelings is in sharp opposition to French citizens. As an illustration, French citizens show high propensities to express their feelings toward the government, through demonstrations and strikes. A survey conducted between 2009 and 2013 revealed that France had the second highest rate of strikes, among 12 European countries, whilst the UK was in the 9^th^ position (Osborne, 2016). Those drastically different approaches to self-expression are in line with work on the Déjà vu experience. Fortier and Moulin (2015) report French citizens’ tendency to describe their experience of “Déjà vu” as more disturbing than English citizens. This corroborates the idea of wider self-expression from this population. Nevertheless, those elements of explanation are exploratory and further scientific investigations should be conducted in order to clarify this finding.

Finally, confirmatory factor analyses were carried out to provide an overview of the complex relationships between the key features of mind-wandering experiences. Our theoretical model hypothesised that the combination of the negative construct (i.e., rumination, depressive symptoms and negative affect), the self-related construct (i.e., future-self thought frequency, self-reflection and rumination), the attentional construct (i.e., mindfulness abilities and cognitive failures) and age would drive the variation of mind-wandering frequency. Within this model mind-wandering frequency is portrayed as an umbrella phenomenon that integrates a multitude of features. Notably, age was found to have a limited contribution to mind-wandering, which is surprising considering the well documented age-related decrease of mind-wandering (Giambra, 1989; Jackson & Balota, 2012; Jackson et al., 2013; McVay et al., 2013). However, the factor of age was highly connected to the self-related construct, a factor composed of two measures known to decrease with age (i.e., rumination and future-self thoughts). In addition to age, the negative construct also presented limited contribution to mind-wandering variation in frequency. Evidence has clearly outlined the relationship between mind-wandering and negative affect (Poerio et al., 2013) and depression or related symptoms like rumination (Smallwood et al., 2005). Research suggests that these negative features are largely found for past related thoughts (Smallwood & O’Connor, 2011), while future related thoughts show positive outcomes such as improved mood and reduction of stress (Engert et al., 2014; Ruby et al., 2013). Critically, mind-wandering experiences have been described as largely future oriented (Baird et al., 2011). Therefore, the limited contribution of the negative construct may reflect the smaller propensity of negatively toned and past-related thoughts. Additionally, a strong relationship was evident between the negative and attentional construct, suggesting that part of the negative construct’s contribution was driven by attentional lapses. Lastly, despite the reasonably good fit of the model to the data, further statistical analyses demonstrated a different fit for both English and French native speakers.

Considering both groups separately, for the English native speakers mind-wandering frequency largely relied on the self-related construct with no direct influence from the attentional construct, the opposite pattern was demonstrated for the French native speakers. These two constructs are shown to be highly related to each other in both models. However, in the British model the self-related construct was composed of ruminative and future-self thoughts, while it was mainly future-self thoughts in the French model. Here we suggest that the contribution of the self-related construct in the British population is driven by their higher rates of ruminative thoughts. Despite no cultural difference outlined on the attentional measures (i.e., mindfulness and cognitive failures) the influence of this construct was significant for the French model. Research clearly states the importance of executive control to flexibly manage ones’ thoughts (Smallwood & Schooler, 2015). This raises questions as to why the attentional characteristics are not central to mind-wandering frequency in the British population. One avenue to consider is the possibility of an indirect effect, where the attentional construct contributes to mind-wandering frequency via its strong relationship with the self-related construct. Subsequently, the factoring of self-reflection into the self-related construct for the British population was in agreement with the previously discussed cultural differences on self-centred thoughts. However, in the French population, where reflection rates are high, this measure contributed to the self-related construct and to mind-wandering frequency in a direct manner. Ultimately, together, these findings demonstrate an influence of culture on the complex experience of mind-wandering.

Nevertheless, some limitations must be taken into account when interpreting these findings. Firstly, conducting an online survey most certainly influenced the characteristics of the population recruited. This is especially true for older adults as not all the aging population has access to internet or is comfortable with the use of electronic devices. Additionally, it provoked an unbalanced distribution of participants across age groups. Secondly, it is difficult to know if the findings differentiating French from English native speakers can be attributed to a cultural difference, or merely a language effect via the interpretation of the questionnaires. Indeed, even if back-translation of questionnaires is commonly used, it does not guarantee equivalence across two languages. Indeed, one word in Language A may correspond to multiple words with slightly different connotations in Language B. For example, a neutral word in English, when translated into French can have a slight negative connotation and therefore would induce differences in participants’ report. Nevertheless, correlations of similar weight are found between questionnaires for each population. For example, depressive symptoms, which were not influenced by culture, correlated positively, at the same extend for both group, with rumination rates (*r* = .58 and *r* =.55), with negative affects (*r* =.64 and *r* =.54) and mind-wandering (*r* =.39 and *r* =.43), which all varied depending on cultural affiliation. Additionally, a study controlling for language of testing evidenced cultural differences between European American and Chinese (Ji, Zhang, & Nisbett, 2004). They stressed that the cultural effect was bigger when language was not taken into account, meaning that language of testing does modulate results but it does not mediate them. Therefore, even though language of testing may lead to changes in responses, such changes occurred in a limited range and do not necessarily threaten the conclusion pertaining to cultural comparisons.

In conclusion, findings from this study clearly evidence cultural differences between English and French speakers, and most importantly they seem to significantly impact one’s experience of mind-wandering. Those results also gave a warning toward overgeneralization of findings. Indeed, if mind-wandering is experienced in every culture, supported by shared cognitive processes, the experience in itself may be different, as highlighted by our findings. First mind-wandering frequency decreased at an earlier age for French speakers. Then, rumination and reflection rates were different in both populations and exerted different age-patterns. Finally, French speaker showed heighten mood as compared to English speakers. Therefore, results stress the importance of considering and measuring cultural affiliations during participants’ recruitment as it can modulate the findings. This study is the first to directly investigate cultural discrepancies in mind-wandering and associated features, therefore, giving the ground work for future research. Future work should move on to laboratory based study and fully measures the socio-cultural affiliation of participants to enable a deeper understanding of cultural influences on mind-wandering experience.
